# Employing the arts for knowledge production and translation: Visualizing new possibilities for women speaking up about safety concerns in maternity

**DOI:** 10.1111/hex.12660

**Published:** 2018-01-17

**Authors:** Nicola Mackintosh, Jane Sandall, Claire Collison, Wendy Carter, James Harris

**Affiliations:** ^1^ Department of Health Sciences University of Leicester Leicester UK; ^2^ Department of Women and Children's Health School of Life Course Science, Faculty of Life Sciences & Medicine King's College London London UK; ^3^ Women's Art Library Goldsmiths London UK; ^4^ Florence Nightingale Faculty of Nursing, Midwifery and Palliative Care King's College London London UK

**Keywords:** arts‐based methods, co‐production, knowledge translation, maternity, patient involvement, speaking up for safety

## Abstract

**Objectives:**

This project used animated film to translate research findings into accessible health information aimed at enabling women to speak up and secure professional help for serious safety concerns during pregnancy and after birth. We tested as proof of concept our use of the arts both as product (knowledge production) and process (enabling involvement).

**Background:**

Emergencies during pregnancy and birth, while unusual, can develop rapidly and unexpectedly, with catastrophic consequences. Women's tacit knowledge of changes in their condition is an important resource to aid early detection, but women can worry about the legitimacy of their concerns and struggle to get these taken seriously by staff.

**Design:**

Arts‐based knowledge translation. A user group of women who had experienced complications in the perinatal period (n = 34) helped us develop and pilot test the animation. Obstetricians and midwives (15), clinical leads (3) and user group representatives (8) helped with the design and testing.

**Findings:**

The consultation process, script and storyboard enabled active interaction with the evidence, meaningful engagement with stakeholders and new understandings about securing help for perinatal complications. The method enabled us to address gender stereotypes and social norms about speaking up and embed a social script for women within the animation, to help structure their help seeking. While for some women, there was an emotional burden, the majority were glad to have been part of the animation's development and felt it had enabled their voices to be heard.

**Conclusion:**

This project has demonstrated the benefits of arts‐science collaborations for meaningful co‐production and effective translation of research evidence.

## 1 INTRODUCTION

While the potential role of patients to contribute to their safety was acknowledged in *To Err is Human*,^1^ until recently, patient safety was largely constructed as a technical and professional issue.^2^ Increasingly, there is recognition of the relatively untapped resources patients and families provide in terms of their collective capacity to provide continuous vigilance over both the patient's health condition and the care that is given.^3^ Research has highlighted the credible and important, albeit often invisible role patients and families play in escalation of care for life‐threatening conditions.^4^

This is particularly pertinent for maternity services, which occupy a high profile, high stake position within the health service.^5^ While for the most part, pregnancy and birth are a normal physiological process, emergencies develop rapidly and unexpectedly with the potential for serious outcomes for mother and/or baby. Delayed diagnosis and treatment of complications such as pre‐eclampsia and reduced foetal movements offer opportunities for the reduction of avoidable harm.^6^ Beyond the loss of life, adverse outcomes include maternal depression and financial costs to parents as well as long‐term economic costs.^7^

Policy and practice guidance includes recommendations to empower women to self‐care, strengthen families’ collective capacity to provide continuous vigilance over women's health conditions and community level programmes to foster safety related behaviours.^3^, ^6^ Online health education campaigns have been implemented aimed at informing women about early warning signs of perinatal complications and appropriate help‐seeking behaviour.^8^, ^9^ Information on websites varies widely,^10^ but tends to provide condition‐specific signs and symptoms.

Greater criticality is needed to assess intended and unintended consequences of health information messaging for women.^11^, ^12^ Health information often assumes a patient knowledge deficit, reinforcing hierarchies and directing recipients to benefit from medical expertise, rather than reinforcing patients’ own expertise and tackling the power differentials that undermine user‐provider relationships.^12^, ^13^ Health messages may also increase women's anxiety about indeterminate risk and undermine trust in their own bodies and the expertise of health professionals.^14^ Enabling effective contribution from patients and family members depends on a complex interplay of personal factors, lay and professional encounters, and contextual influences. Reports continue to highlight that women have their reports of safety concerns dismissed by staff with devastating consequences for mother and baby.^15^, ^16^, ^17^

This study reports on the *Re‐Assure* project which tested as proof of concept whether arts‐based methods could effectively help us translate our research findings on women speaking up about their serious safety concerns into a useful health information resource to help women as they journeyed through the maternity system. Active interaction of art with research evidence can effect change by directing critical dialogue towards social issues.^18^, ^19^ It can highlight features of lived experience otherwise ignored.^20^ Using art for knowledge translation privileges insider knowledge and is efficient at imparting complex ideas in accessible and meaningful formats, but without oversimplification.^21^, ^22^ We evaluate our use of the arts both as product (ie production and dissemination of the animation) and process (ie public and professional engagement in the project and their assessment of whether the product was rendered convincing by the reality of practice).

## 2 THE RE‐ASSURE PROJECT

### 2.1 Designing the project brief, aims and objectives

Our previous programme of research explored the management of complications in maternity^23^ and highlighted the credible and important role women played in escalation of care, but also the difficulties experienced in enacting this role in practice.^24^, ^25^ Findings from this research demonstrated how women often relied on family members to ensure their safety. The nature of women's safety concern, how it was expressed, existing relationships with staff, their previous experiences of health services, and language influenced women's ability to contribute to escalation of care.^26^

Funding for the Re‐Assure project was received for 6 months (July 2016‐January 2017) as part of the Cultural Institute at King's Arts, Health and Wellbeing scheme. The scheme brought together health academics at King's and artists with experience of working in the health‐care sector to brainstorm arts‐based solutions to health‐care problems. Seed funding was awarded to the best proof of concept ideas to emerge.

**Figure 1:**
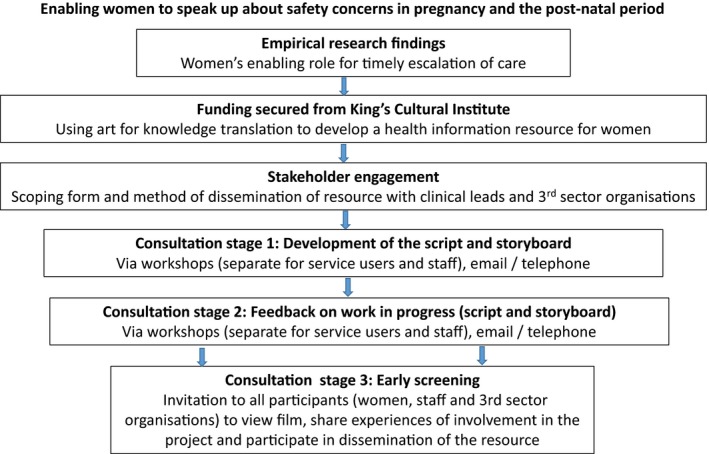
The re‐assure project: engagement and consultation

The project team consisted of researchers with social science (psychology and sociology) and clinical (midwifery and critical care) backgrounds, and a cultural partner (writer, artist and facilitator). Figure 1 provides details of the engagement and consultation processes we employed to develop the project brief, process and end product.

We developed the project brief following conversations with maternity leads from Guy's and St Thomas’ NHS Foundation Trust and representatives from Sands (Stillbirth and Neonatal death charity) and Tommy's (a charity that funds research into miscarriage, stillbirth and premature birth, and provides support and information about healthy pregnancy for parents‐to‐be), and in line with the strategic priorities of NHS England and the London Maternity Strategic Clinical Network to reduce avoidable maternal death, and stillbirth rates. Based on our research findings, we aimed for the health information resource to (i) legitimize women's lay expertise and intuitive sense when something was wrong, (ii) provide information about “red flag” symptoms which could signify a serious underlying condition, (iii) signpost what action women need to take in terms of help seeking and (iv) help women structure their calls for help and have an understanding of what response to expect from health‐care professionals.

We chose animation with an embedded script to deliver the health messages because of its ability to permit the exploration of difficult issues in a non‐threatening form during its creation.^19^ Health messaging offers potential for behaviour change via techniques including prompts, rehearsal, modelling, social processes of encouragement, pressure and support, and providing information about health consequences.^27^ Animation as a product can also enable dissemination via various modes of delivery. We aimed to maximize reach to women by designing the animated film for screening in waiting rooms and clinics across primary and secondary care settings. It would be played on a loop, in multiple languages, with key images and messages held as “stills,” providing links to further information and resources. We also aimed for the animation to be disseminated via social media and online.

### 2.2 The consultation process

As this project consisted of public consultation in the design of a health information resource, ethical approval and consent were not required. Sands and Tommy's helped us develop a call‐out (see Table 1) for women who were delivered via their social media platforms (Facebook and Twitter). We were keen to work from women's own definitions of “serious complications” and their own sense of agency in recognition and help seeking, rather than imposing professional frames of reference. We adopted an inclusive approach to time since birth, as women's memories of childbirth remain strong over time.^28^ Three other voluntary and community or “3rd sector” organizations who provide information resources for pregnancy complications (Action on Pre‐Eclampsia (APEC), Mama Academy and Intrahepatic cholestasis of pregnancy (ICP) support) also posted the call‐out via their social media platforms.

**Table 1:** Re‐assure call‐out via social media

Did you experience serious complications in pregnancy or the postnatal period?
Did you recognise that something was wrong? Did you feel able or unable to seek professional help for your concerns?
If so, can you help us to develop a film to help women (and family members) spot early when they are becoming sick and work with staff to get the right help quickly?

We worked with senior midwifery and obstetric leads at the participating Trust to design a flyer to recruit staff to help with the project. This was emailed out to all staff working in maternity services including support workers, midwives, obstetricians and neonatologists.

We included three consultation stages for developing and testing the animation. The first to help us develop the script and storyboard, the second to receive feedback on the work in progress, and the third to hear feedback on the finished product and participants’ experiences of involvement during the project. We ran separate workshops for service users and providers for the first two stages. We also took advice from Sands and for the first stage, organized a separate workshop for women who had experienced the death of their baby before, during or soon after birth. A member of Sands staff attended the workshop to provide a means of support. All participants (service users, providers and 3rd sector representatives) were invited to an early screening of the animation. In all three stages, we offered participants the chance to contribute via email and telephone if they were unable to attend the workshops or screening.

The first two workshops for women were designed to be informal and included warm‐up exercises and setting of ground rules and group norms (eg it's ok to get upset or to leave the room; the importance of respecting one another's experience and views). The artist (an experienced facilitator) led the workshops which were also attended by 2‐3 of the researchers. The women worked in facilitated small groups, each creating their own storyboard and visual timeline of events on flipcharts. Common themes were then shared within and across groups. The process was structured around the benefit of the women's hindsight for the development of this resource and focused on timelines (key moments) and strategies the women believed useful for help seeking and navigating the maternity system (see Table 2). We used the same questions when eliciting views from those women who could not attend the workshops but wished to participate via email or telephone. The artist and lead researcher carried out the telephone interviews. We obtained consent from all the participants to record their views via telephone and workshops and to use photography to capture their storyboards for the purposes of quality improvement.

**Table 2:** Structure of the first workshops for women

Warm‐up exercise—turn to person next to you—share an example *from your own broad life experience,* where you have successfully negotiated a tricky situation? This could be anything, large or small (it doesn't have to be connected to health). Tell us what happened.
In small groups of 3‐4
Can you each share your timeline of your maternity experiences, starting with when you recognised there was a problem and what followed.
Can you identify key moments where you were not heard?
*What was helpful in your timeline for getting heard?*
In groups can you collect key hindsight messages, if advising someone in your situation now, what would you tell them?

The first consultation process with staff also involved an informal workshop with small group work which involved asking them to identify their top “red flags.” We asked them in their groups to write down on flash cards their experiences of what was helpful to hear from women, and what usefully staff could do or say to help overcome barriers to women and family members sharing safety concerns.

The artist thematically analysed the transcripts and narratives, and worked with the visual representations and flashcards to look for recurring narrative ideas, relationships, linguistic and rhetorical devices (eg metaphors and images),^29^ and how best to represent the data in terms of the animation's composition, form and emotional tone.^30^ Early ideas for a storyboard and script were presented to the team which led to further iterative refinement.

The second stage involved presenting the draft storyboard and script to participants (users and staff) at two workshops. We emailed the script annotated with key images to those who were unable to attend. We collected verbal and written feedback on the drafts (content, imagery, anything missing) which triggered further thematic analysis and team discussion, and ultimately further refinement of the script and storyboard. Finally, the third stage involved a screening of the final film. All participants were invited to view the animation online and/or attend an early screening prior to its dissemination, providing opportunity for stakeholder feedback on the animation's perceived usefulness.

### 2.3 Involvement

Sixty‐five women registered their interest and 34 women participated in the project. For the first two consultation stages, 14 (38.9%) attended a workshop, 14 (38.9%) were consulted by email and 8 (22.2%) participated by telephone (two women used both email and telephone). Thirty (88%) of the women gave feedback on the draft story boards and 23 (68%) gave feedback on the finished animation.

Of those that participated, 23 had experienced pre‐eclampsia and/or eclampsia, 14 a pregnancy or neonatal loss, 2 had traumatic births and one each had a severe post‐natal mental health condition, obstetric cholestasis, premature birth and neonatal infection. Thirty of the women provided demographic data for us (see Table 3).

**Table 3:** Service user demographic data

Age	
25‐34	5
35‐44	13
>45	12
Self‐defined ethnicity	
White (British, Irish, European)	25
Black and minority ethnic (British, Asian, African, Caribbean, Latin American)	4
Non‐response	1
Language	
English first language	29
Other first language	1
Education	
School/college	2
Undergraduate degree	12
Postgraduate degree	15
Non‐response	1

In terms of staff, one support worker, nine midwives, five obstetricians and three senior managers participated in the first two consultation stages, as did eight representatives from the participating 3rd sector organizations. We received feedback on the finished animation from 12 staff members (10 midwives, 1 obstetrician and 1 senior manager), 3rd sector organization and maternity services liaison committee representatives (n = 6).

We collected written feedback from women on their experiences of involvement in the project, both at mid‐point (after the second consultation stage using an online anonymized survey tool [Survey Monkey—see Appendix]) and at the end of the project (at the screening and via email), asking women to share their perceived contribution to the making of the film, and their experiences of involvement. We drew on 4Pi31, national involvement standards^31^ which provides a broad, inclusive framework by which to understand the effects of involvement.^2^ Twenty‐nine (85%) women participated in the mid‐point survey and 23 (68%) gave written feedback at the end of the project.

## 3 FINDINGS

We consider firstly how our use of arts‐based methods enabled engagement with the research evidence to shape the production of the animation. Secondly, we reflect on the involvement process for the women participants.

Employing methods of cultural production and learning from the benefit of our participants’ hindsight brought the social and material into relation with one another. It enabled us to move from our previous research findings (what we had learnt from women's prior experiences) to new forms of knowledge construction (insights into “what could be” ie opportunities for change). Gen, the protagonist in our animation, represented the multiplicity of women involved in our project. Five themes from our original research were further developed into opportunities for change: developing a resource for complications; the power of self‐diagnosis; advocacy through family and friends; the lay‐professional team; and the value of a social script for speaking up.

## 4 CONSULTATION STAGE 1: DEVELOPMENT OF THE SCRIPT AND STORYBOARD

### 4.1 Gen, representing the multiplicity of women

We chose to create a single central figure, “Gen” (“generic”), as the protagonist for the animation to bring together the multiplicity of women who were involved in our original research and the Re‐Assure project. Gen (coloured green) assumed a symbolic ethnic and age neutral status. We realized through the consultation process that the animation needed to be affirmational, validating Gen's lay expertise, and her authority to manage her own maternity journey. We also heard that the narrative needed to explicitly acknowledge unpredictability and clinical uncertainties in Gen's journey. This was structured into the script; “no matter how carefully you prepare, you can't guarantee what will happen” and “your journey may be straightforward but it may not be.” We designed the script to be action‐oriented, sharing resources and advice from “women who have been there.”

### 4.2 Developing a resource for complications

Our original research highlighted that women's discussions with health professionals tended to be orientated around plotting points and verifying progress in the antenatal period rather than around their health needs and safety concerns. Women's expectations of pregnancy and childbirth, and social norms also help reinforce a silencing form of “verbal asepsis.”^32^ We were advised by users and professionals that the animation needed to explicitly acknowledge that complications *do* occur for some women, together with the associated stigma of not sailing through the “perfect pregnancy.”

Rather than drawing on wider meta‐narratives of danger and positioning women as vulnerable to a host of adverse conditions,^33^ we framed the animation as a resource *in case* this happened.

### 4.3 The power of self‐diagnosis

We had originally planned to include “red flags” of impending critical perinatal illness in the animation to aid recognition and response. However, through working with our women participants, we realized that representation of these red flags in the storyboard threatened to exclude those women who did not present with them and provide false reassurance for women without these specific symptoms. Our research findings had demonstrated significant variability in timelines, severity, presentation and interpretation of signs and symptoms for women presenting with maternity complications and this was reaffirmed by the women we consulted with. The staff workshop also highlighted the plethora of signs and symptoms perceived by midwives and obstetricians to qualify as “red flags,” and variation in individual professional's interpretations as to their significance. We therefore designed the animation to refer to generic safety concerns rather than being bound by clinically defined pregnancy and post‐natal conditions.

Our previous research had demonstrated that, while lists of early warning signs can act as structuring devices to aid diagnosis and triage, they also marginalize the complexities of managing risk in maternity care. Technical language and reliance on categorization systems can contribute at times to “missing the zebra” (the exceptions to the rule).^34^ In the animation, we chose to depict a deterioration in Gen's health not by specific technical markers but by the development of generalized stripes across her body (including her head), to illustrate how physical and mental health are intertwined.

The consultation process with both women and staff affirmed that the animation needed to validate women's expertise at self‐diagnosis (even in the absence of “red flags”) and their “intuitive sense” that things were wrong as legitimate markers of a developing serious maternal complication. We shifted the emphasis in the script from red flags to the importance of “knowing *your* normal,” an asset identified through the benefit of women's hindsight. “Knowing *your* normal” reinforced the importance of women tuning into their sense of self (somatic, psychological and cognitive). Women's tacit knowledge about changes in their condition was presented as an important early warning sign to help them work with professionals to detect serious life‐threatening conditions.

Our original findings demonstrated that hierarchies of knowledge and social norms regarding “acceptable patient‐provider behaviour” acted as barriers to participatory action in escalating care in the perinatal period. Many system level issues such as discontinuity of care and brief time‐pressured maternity care consultations reinforced status differentials between maternity users and providers. The consultation process helped us to use the script to reposition women as knowledgeable about their own bodies and minds, and strong in terms of able to express fear and vulnerability which is an important component to problem identification and help seeking. Our women participants helped us choose appropriate vernacular to confront gender stereotypes, for example “not wanting to be an annoying first time Mum” and a “time waster.” Inclusion of feedback from the staff workshop that “we would rather reassure you a 100 times rather than miss spotting a problem once” provided professional endorsement for the significance of women's help seeking for reducing avoidable harm.

#### 4.3.1 Family and friends as advocates

Our previous research data highlighted that family and friends were often able to help with picking up changes in their condition, and at times of vulnerability, could speak up and act as powerful advocates for women. Recognition of this additional resource was therefore built into the animation. We did receive feedback from a few of the women we consulted that some partners and social networks had provided them with false reassurance. We therefore tried to reinforce the importance for women to trust their own sense of safety and to seek help from those who could effectively act as an advocate for them.

#### 4.3.2 The lay‐professional team

There was widespread agreement amongst our participants that the animation needed to emphasize how both women *and* staff needed to work together to enable timely recognition and response. Perceived power imbalances and sense of difference between staff and women were addressed through imagery (both appearing on the same level, both having a dog as a companion). Inclusion of staff in the production process helped to legitimize the messaging. The animation needed to emphasize the importance of diverse knowledge and lay understandings for diagnosis, while not undermining women's trust in staff and the maternity system.

### 4.4 The value of a social script for speaking up

Using the benefit of their hindsight, both users and providers helped us build a social script to add to the animation to enable women to get heard**.** This included “make a list of your concerns,” “Begin by saying ‘I am concerned’…” and “Be specific—what has changed?” Importantly, it acknowledged that many women in our previous research and our participant group had had their concerns dismissed by staff and felt unsure what to do next. We built into the script the message “If you can't make yourself heard ask for a second opinion.” This aimed to legitimize the importance of organizations having a response system in place for women to turn to if they had lost trust in the staff looking after them.

## 5 CONSULTATION STAGE 2: FEEDBACK ON THE WORK IN PROGRESS

We had largely positive responses to the draft storyboard and script from the user group, staff and 3rd sector representatives. We took on board their suggestions how to improve content and delivery. We took the majority view when opinions were divided, for example how serious to make the messaging and whether to add in any technical details about particular maternity complications. A number of women noted the effectiveness of this resource was crucially tied into staff ability to listen and value women's contributions, reinforcing the need for us to involve staff in dissemination. They also emphasized the importance of using social media for dissemination. Tommy's charity identified that they would be keen to potentially work with us in a follow‐on project to develop a shortened version of the resource for social media.

## 6 CONSULTATION STAGE 3: EARLY SCREENING AND DISSEMINATION

The film received positive feedback from our user group, staff and stakeholders, and is now publicly available (Table 4).^35^ The majority perceived that the animation had tackled issues of power and stigma, it had made explicit the importance of a woman's innate knowledge and intuition for early detection of complications, and had usefully drawn on women's experiences for the benefit of others. Our stakeholders have helped us with dissemination. We have worked with Guy's and St Thomas’ NHS Foundation Trust to develop 4 language translations (French, Spanish, Portuguese and Polish) for their triage settings. Following the early screening, we worked with Tommy's charity to develop a shortened version of the Re‐Assure animation for social media which was launched as part of the “Always Ask” campaign.^36^ The campaign was endorsed by the Royal Colleges (RCM and RCOG) and by NHSE and was supported by Sands, ICP Support, APEC, Mama Academy via their websites, twitter and social media. In the first week, the shortened film had over 250k views, over 2k shares, and reached over 1million. Both the original animation and the shortened version are hosted on Tommy's website with links to other resources on pregnancy complications.

**Table 4:** Feedback from participants on the finished animation

	Feedback
Tone and imagery	The film is warm and reassuring and coming from a medical background, the message feels really earnest and genuine. It would have given me a lot of confidence [User]
	I think the tone is good—reassuring rather than scary. The music helps [User]
	I thought that the graphics were greatly improved, and liked the list of empowerment strategies [User]
	We feel the stripes analogy work well—some of my symptoms didn't fit Emma's diary description—so we both brushed them off! [User]
	I think the film is quite “punchy” and easy to watch with some strong messages. The points about asking for help, getting others to support you and particularly going back in the door were very strong. I liked the shield with baby on board and being assertive for your baby. I particularly like the fact that it is uncluttered [Midwife]
	Clear, simple, reassuring messages [Senior Manager]
	I was so excited to see this video as it accords wholly with the messages that we are constantly getting from women [3rd sector organisation representative]
Importance of key messages	I think that the primary message—if things don't feel right trust yourself, tell a professional—is valuable [User]
	I thought it was great—I think the tag line “You are the expert on you” really sums it up [User]
	I think the “Listening to YOU” was the part that was the most relevant for me. The Gut Feeling that something is wrong and to act on it [User]
	The two main points (that stood out) for me were to push for clarification/trust your instinct and the notion that raising an issue doesn't make you a time waster or someone who fusses over nothing [User]
	I think the one thing that is missing is the concept of time. So particularly with reduced fetal movements, the number of times women say “oh I knew I was coming to see you so I didn't bother going in” [Midwife]
Utility	We feel the film would have made us feel more at ease with seeking advice when we were worried [User]
	I think if I'd have seen this film before my daughter's birth then I would have been more confident in raising my concerns with hospital sooner, therefore possibly avoiding some of the complications we suffered. It would of also of helped me after the birth to get the answers that I needed [User]
	The film is good and I'm pleased to see it out there, I hope it goes far and wide. I think things have got better in the last 6 years but I did raise concerns and my midwife minimised them so it's really the midwives that need some educating as well [User]
	The film will give women confidence to report any problematic symptoms, ask for a second opinion if they want one and not to feel embarrassed by reporting the same problem again after gaining reassurance [3^rd^ Sector Organisation representative]
	I can see me using this film a lot. It will help those women in our facebook groups who find it hard to keep going back and asking for what they need. We do everything we can to support them and we provide them with the evidence but this film will really help emphasise that it's okay to be keep going back [3^rd^ sector organisation representative]
	I would certainly show the film to others and feel it reflects much that was discussed in the workshop [Midwife]
Value of the guide “How to ask for help”	The QUESTIONS TO ASK section is excellent. How often do we ask a question and then later forget the answer. Writing it down and also planning a few questions to ask is a superb tip. The journey…. changing direction is also very good [User]
	I liked the list of empowerment strategies. This would not have helped me in my vulnerable state… but would be of more use for those women whose decline is noticed by themselves, and family and friends [User]
	I've done a bit of caseloading this year and there was an occasion when I told a woman to go into triage to be assessed (she'd been sent a bit round the houses), and I told her to start with “I am concerned” so she would be listened to [Midwife]

## 7 THE INVOLVEMENT PROCESS

We received feedback after consultation stage 2 that our methods of consultation and involvement were acceptable for the majority of women (see Table 5). Two women selected the negative descriptors “excluded” and “discouraged” from the fixed choice list, while also selecting positive descriptors such as “pleased,” “taken seriously” and “glad” to be a part of it. After the film production, we heard that the majority of women who participated in the project found the involvement process meaningful and worthwhile (Table 6).

**Table 5:** Interim feedback on the involvement process

Survey question	Response categories	Responses
How acceptable were the online recruitment methods we used to invite you to take part in the project?	Extremely acceptable	18 (62%)
	Very acceptable	11 (38%)
	Somewhat acceptable	0
	Not so acceptable	0
	Unacceptable	0
How clearly did we explain the aims of the project?	Extremely clearly	16 (55%)
	Very clearly	13 (45%)
	Somewhat clearly	0
	Not so clearly	0
	Not at all clearly	0
How clearly did we explain your role in contributing to the animation production?	Extremely clearly	15 (52%)
	Very clearly	14 (48%)
	Somewhat clearly	0
	Not so clearly	0
	Not at all clearly	0
How comfortable did you feel sharing your maternity experiences via email, telephone or workshop?	Extremely comfortable	15 (52%)
	Very comfortable	9 (31%)
	Somewhat comfortable	3 (10%)
	Not so comfortable	2 (7%)
	Not at all comfortable	0
How involved have you felt in the development of the script and storyboard to date?	Extremely involved	9 (31%)
	Quite involved	11 (38%)
	Somewhat involved	4 (14%)
	Not so involved	4 (14%)
	Not at all involved	1 (3%)
How well has the project taken on board those maternity issues and concerns that are important to you?	Extremely well	10 (36%)
	Very well	16 (57%)
	Somewhat well	2 (7%)
	Not so well	0
	Not at all well	0
	Non‐response	1

**Table 6:** End of project feedback on involvement process

	Feedback
Feeling heard in the consultation process	I can definitely see things that I mentioned being brought out, as well as some of the things others in my group said too [User]
	I definitely feel that my contribution has been reflected and honoured, and I certainly feel that my involvement has been worthwhile [User]
	I think it's brilliant, it's clear you listened to all our views when putting it together. I certainly feel it was worthwhile being involved [User]
	Thanks for giving us such a powerful voice and staying true to our message which must have been such a huge responsibility [User]
	Acknowledgement that our opinions matter and for you to hear our story [User]
Contributing to a worthwhile resource	By sharing my experience, I hope it can go to help women during their pregnancy and give them the confidence to speak up if they feel that something may not be right, no matter how trivial it may seem [User]
	The thought of being able to help others and perhaps potentially prevent stillbirths/neonatal death is somewhat comforting [User]
	The hope that this may help someone else have the confidence to speak out when they are worried makes this worthwhile [User]
	I hope it has in some small way helped to produce what looks to be a very valuable resource that I believe could prevent or reduce serious adverse outcomes for mothers and babies [User]
	To know that my own experience could help other people [User]
	To have our concerns and experiences acknowledged and used to inform future care is invaluable. If even one baby death is prevented it will have been worthwhile [User]
Personal positive experience of involvement	I'd forgotten how powerless I felt at the time and it's great to be able to contribute towards something that may help to improve others’ confidence in raising awareness and any concerns or worries they may have during pregnancy/childbirth. Incredibly valuable exercise [User]
	The experience was a great relief to me, I had not realised how much I needed to make my peace with what happened [User]
	It has allowed me to take back a bit of control—not necessarily of what happened (as that is in the past), but of how I want to move forward (positively). Making me an active participant, trying to help others. Not a victim role. It has been massively helpful to do this, thank you [User]
The emotional cost of involvement	It felt a safe and secure environment. More painful emotionally than I had expected in terms of remembering how I felt—yet on reflection also cathartic [User]
	I found the film upsetting as I wish I'd seen this when I was pregnant as I might have pushed my concerns and he'd be alive today. I guess that means you're getting it right though and if it saves one baby it's worth it [User].
	It is always difficult to revive distressing experiences, but the format managed this as well as anyone could expect [User]
	It's very emotional to discuss and takes you right back into those experiences [User]
	Talking and re‐living my experience was very emotional for me, more than I had anticipated. I would still do it again though. I know now that emotion is not something to be ashamed of that the experience I had will always be there and feeling the way I do about it, is OK [User]

Perceived benefits were linked to the experience of taking part and having their views taken seriously, the opportunity to take back some control and to use experiential knowledge to help others, and belief in the value of the animation itself. A number of women also shared the difficulties they experienced with participation which largely centred on the emotional reaction to reliving experiences. One woman described finding it difficult to hear that little had changed since her pregnancy, highlighting how the workshops acted as social spaces and positioned women's individual experiences amongst others. While these women reported there was an emotional burden associated with participation, this was offset by the cathartic experience of sharing and being heard, and being able to contribute to a resource that has potential to help others. The majority of our user group (including those that found reliving their experience difficult) have expressed interest in working with us on future research.

## 8 DISCUSSION

Our project demonstrates how participatory arts‐based methods are an effective means to facilitate knowledge production and translation.^37^ We conceptualize knowledge as both a social process and a resource for sense‐making and action.^38^

Within organizations, including health care, an adverse consequence of the division of labour, hierarchy and specialization is that knowledge becomes segregated, partial and incomplete.^39^ *More* information typically is viewed by staff as the solution to coping with clinical uncertainty. Our staff workshop highlighted the plethora of signs and symptoms perceived by midwives and obstetricians to qualify as “red flags.” Via a process termed “uncertainty absorption,”^40^ technical language and classification schemes serve as a method of reinforcing notions of clinical certainty and marginalizing the complexities of managing risk. Biomedical information tends to be privileged over experiential and embodied, demonstrating the “micro politics of knowledge”^41^ at play. Our research substantiates Greenhalgh et al.'s^42^ insights that the evidence hierarchy inadvertently devalues the patient's experiential contribution to care and reinforces power imbalances that suppress the patient's voice. We produced a potentially useful material and digital resource that privileged women's experiential and embodied knowledge,^43^ and their collective resources and capacities as an aid for other women with serious concerns about their or their babies safety.

An important consideration for this project was the relationship of the animation's visual imagery and text to the research findings being translated. We found that the visual *added* to the textual, and opened up a space for new understandings about speaking up about safety. Art draws the observer into a particular socio‐cultural world with all its textures, sounds, gestures and movements in contrast to textualism, which flattens out the flux of human relationships.^37^

Existing online resources to aid women's help seeking tend to rely on individuals’ accounts of their own experiences.^44^ While these can provide contextual information about causes and consequences and help people to understand what may happen,^45^ they can exclude others if the experiences presented are not shared.^46^ To foster inclusivity, we chose to present a generic meta‐narrative rather than a series of personal stories. The potential disadvantage of us adopting this approach is that meaning and significance could be lost as the script is separated from social context, interaction and technical detail.^47^

Given continuing reports of poor system responsiveness to women and patients’ voices^15^, ^16^ and by aiming the animation at women, we could be accused of diverting attention away from structural inadequacies in the maternity system, and staff's role in listening to women. This was a small‐scale proof of concept project, and we deemed that the women's role in speaking up for safety was a necessary starting point. Encouraging women to seek help for safety concerns from a system already under great strain,^48^ brings with it concerns that the “worried well” may detract resource away from those that require urgent clinical attention. However, this construction of “inappropriateness of demand” perpetuates a particular moral position of blame towards the individual seeking help, while detracting attention away from the system and its ability to respond.^49^ Unless women presenting with “false alarms” are taken as seriously as those presenting with actual emergencies, the potential for addressing delayed recognition and response is limited.

Women experiencing pregnancy and complications for the first time are expected to use their own judgement in determining when it is appropriate to seek medical advice. They are placed in a “double bind” by the sick role in which they are placed^50^ in that they then need to demonstrate their co‐operation with legitimate expertise by deferring to the midwife or doctor's judgement. Any response to the professional's assessment that challenges this asymmetry inescapably undermines the women's grounds for seeking professional help in the first place.^51^ The animation highlighted the importance of organizations offering a response system so that women are able to access a “second pair of professional eyes” as a valuable safety net to help mitigate against errors.^52^ Further research is needed to explore how this might work in practice in terms of making consultations more democratic and addressing power imbalances.

Caution, however, is required to ensure the burden of responsibility is not shifted onto women while failing to address user‐provider hierarchies and power divides around diagnosis.^53^ The high levels of engagement in the project suggest that lay‐professional hierarchies and practices are not necessarily deterministic and that there is opportunity for actors to change and recreate social relations. Grass root support for the animation could leverage some degree of social change, while the animation offers a potential role in staff education and training. In terms of assuring impact, there needs to be widespread commitment from the Professional Colleges and organizations to incorporate this into mandatory training and also to take on board the messages within it. Attention will need to be played to ensure there is sustainability beyond the lifetime of the project.^54^ Further research is required to assess uptake and impact, that is how the animation is used and enacted in practice by women, families *and* staff, and what role it plays in enabling women and staff to work effectively together. Further evaluation could involve surveying women about their experiences using it in practice, assessment of its use in triage settings and staff education and training, and change in women's and staff attitudes to women speaking up about safety.

Viewing film is a patterned social activity shaped by social contexts, cultural conventions and group norms.^55^ While the demographic profile of the sample for our original research was mixed, the women participants for the Re‐Assure project had higher levels of education and were mostly of white British origin. This reflects the typical profile of people involved in patient and public involvement in research^43^ and raises questions about the implications of increasing opportunities for participation when it increases the overrepresentation of the already well represented.^56^ Further implementation research is required to test the methods of dissemination, and the animation's cultural transferability, that is whether storyboard meanings are “shared” and hold significance for women of diverse cultural and language backgrounds.^57^

While our project could be deemed successful in terms of enabling knowledge production and translation, it was not without its challenges. We found a fluidity to the boundary between public engagement in the translation of existing research and the generation of what could be termed “new data.” The constraints of time and budget associated with a small proof of concept project imposed limits on our ability to structure in efforts to enable meaningful participation. We were only able to overcome these limits by investing substantial amounts of our own time and resource. Project management involved a significant amount of boundary work; managing boundaries between art and science, and theory and practice, with their associated differences in epistemologies, ontologies and methods. Team members’ personal belief in and commitment to the project helped with mediation of differences. These insights demonstrate that, for multiknowledge conglomeration or *bricolage* 58 to occur, sufficient supportive infrastructures need to be in place, and mechanisms need to be set up early on in the project to foster open dialogue, trust and shared understandings (eg via a locally agreed concordat).^59^

For those women that did participate, within the democratic tradition, involvement is seen as something that has intrinsic value in and of itself.^2^ We employed a number of strategies to level the power differentials that accompany the co‐production process. The artist led the workshops and to some extent acted as a boundary spanner, mediating professional and lay differences in lay and academic knowledge.^58^ Our use of women's own definitions of serious complications and generic narratives for the animation rather than the selection of individual case histories appeared to foster inclusivity. The design of our small group workshops, individual email and interview encounters appeared to enable the majority of women to feel heard. However, as researchers, we must also accept the responsibility of holding harm, particularly for the participants who expressed adverse feelings.^60^ Because it heightens the senses, using arts‐based methods may increase the likelihood of making an impact (whether negative or positive) on those involved.^61^ Assessing unintended as well as intended consequences of the consultation process is key, as is sharing of useful strategies to mitigate associated risks.

## 9 CONCLUSION

Our use of arts‐based methods enabled active interaction with research evidence and development of a resource considered useful and meaningful by stakeholders. Our women participants, staff, 3rd sector representatives and strategic leads remained engaged during the duration of the project and have committed to continue to support dissemination of the animation. Future research will need to examine ongoing levels of engagement by women through grassroots, community and 3rd sector networks, and staff via professional communication channels such as the Royal Colleges and NHS bodies. The potential impact of the animation on women and babies’ health and social outcomes is tied up with changing *both* women's and professionals’ attitudes and behaviour. This in turn requires a redistribution of the balance of power and a recognition of the legitimacy of lay *and* professional knowledge/expertise for improving perinatal safety and how broader shifts in patients’ and providers’ attitudes to power and knowledge are required.

## CONFLICT OF INTERESTS

None declared.
