# Supplementary material for: Employing the arts for knowledge production and translation: Visualizing new possibilities for women speaking up about safety concerns in maternity
Source: Health Expect. 2018 Jan 17;21(3):647–58. doi: 10.1111/hex.12660 (PMC5980615; doi:10.1111/hex.12660)
Supplement: Supplementary file 1 [file HEX-21-647-s001.pdf]

1. Your feedback is important to us

We would really like to hear your experiences of taking part in the Re-Assure project. We'd love to hear from you about our recruitment methods, and how meaningful it has felt to participate in the development of the animation. This will help us learn how best to involve women in future research studies. The survey should only take 5 minutes, and your responses are completely anonymous.

The survey will close on 21st October 2016.

If you have any questions about the survey, please email us: nicola.mackintosh@kcl.ac.uk

We really appreciate your input!

1. How acceptable were the online recruitment methods we used to invite you to take part in the project?

- ◯ Extremely acceptable
- ◯ Very acceptable
- ◯ Somewhat acceptable
- ◯ Not so acceptable
- ◯ Unacceptable

Any comments?

2. How clearly did we explain the aims of the project?

- ◯ Extremely clearly
- ◯ Very clearly
- ◯ Somewhat clearly
- ◯ Not so clearly
- ◯ Not at all clearly

Any comments?

3. How clearly did we explain **your role** in contributing to the film production?

- Extremely clearly
- Very clearly
- somewhat clearly
- Not so clearly
- Not at all clearly

Please add any comments

4. How did you share your maternity experiences with the project team? Please tick all that apply

- via workshop(s)
- via telephone interview with Claire or Nicola
- via email

5. How comfortable did you feel sharing your maternity experiences via email, telephone or workshop?

- Extremely comfortable
- Very comfortable
- Somewhat comfortable
- Not so comfortable
- Not at all comfortable

Any comments?

6. How involved have you felt in the development of the script and storyboard to date?

- Not at all involved
- A little involved
- Somewhat involved
- Quite involved
- Extremely involved

Any comments?

7. Having seen the draft storyboard, I feel (please select as many of the words as you like)

- [ ] Heard
- [ ] Unheard
- [ ] Taken seriously
- [ ] Not taken seriously
- [ ] Pleased
- [ ] Unhappy
- [ ] It's been worth the effort
- [ ] It's been a waste of time
- [ ] Represented
- [ ] Misrepresented
- [ ] Optimistic
- [ ] Pessimistic
- [ ] Glad to be a part of it
- [ ] Sorry to be a part of it
- [ ] Included
- [ ] Excluded
- [ ] Reassured
- [ ] Discouraged
- [ ] Excited
- [ ] Indifferent
- [ ] Disappointed
- [ ] Satisfied

Other (please specify)

8. How well has the project taken on board those maternity issues and concerns that are important to you?

- ◯ Extremely well
- ◯ Very well
- ◯ Somewhat well
- ◯ Not so well
- ◯ Not at all well

Please comment - we would love to hear your views

9. What, if anything, has been the value in participating in this research?

10. What, if anything, has been difficult about participating in this research?

2. Background information

To help us understand a few background details about the participants that have contributed to the project we would really appreciate it if you were to give us a few background details. The information will be kept confidential, and not linked to you personally.

If you have already provided us with these details at one of the workshops you can opt out of the next section

11. Have you already provided background information at one of the workshops?

- ◯ Yes
- ◯ No

3. Background information

12. What is your age?

- 18 to 24
- 25 to 34
- 35 to 44
- 45 to 54
- 55 to 64
- 65 to 74
- 75 or older

13. What is your postcode?

14. Please describe your race/ethnicity.

15. Please describe the highest level of school you have completed or highest degree you have received

16. Is English your first language?

- Yes
- No

Thank you so much for participating in this survey and in the project!

Re-assure – Emboldening women to share their safety concerns about life threatening illness, and enabling maternity response

‘Re-Assure’ is a collaboration between King’s Improvement Science, King's College London’s Women’s Health Academic Centre, and artist Claire Collison, brokered and supported by the Cultural Institute at King’s College London. This work is supported by the National Institute for Health Research (NIHR) Collaboration for Leadership in Applied Health Research and Care South London at King’s College Hospital NHS Foundation Trust.
